# Effects of physical exercise in patients undergoing haematopoietic stem cell transplantation: systematic review and meta-analysis

**DOI:** 10.1007/s00520-025-10194-5

**Published:** 2025-12-02

**Authors:** Carlos Martín-Sánchez, Luis Polo-Ferrero, Mónica Baile-González, Sara Marcos-Asensio, Eduardo José Fernández-Rodríguez, Roberto Méndez-Sánchez, Victor Navarro-López, Ana Silvia Puente-González, Lucía López-Corral, Almudena Navarro-Bailón, Jesus Perez, Juan Luis Sánchez-González

**Affiliations:** 1 Faculty of Nursing and Physiotherapy, Department of Nursing and Physiotherapy, University of Salamanca, Salamanca, Spain; 2 Institute of Biomedical Research of Salamanca (IBSAL), Salamanca, Spain; 3 Hematology Department, Complejo Asistencial Universitario de Salamanca, Salamanca, Spain; 4 Department of Physical Therapy, Occupational Therapy, Rehabilitation and Physical Medicine, Faculty of Health Sciences, Universidad Rey Juan Carlos, Madrid, Spain; 5 Department of Human Anatomy and Histology, University of Salamanca, Salamanca, Spain; 6 Department of Medicine, University of Salamanca, Salamanca, Spain; 7 Department of Neuroscience, Institute of Biomedical Research of Salamanca (IBSAL), Salamanca, Spain; 8 Department of Psychiatry, University of Cambridge, Cambridge, UK; 9 Norwich Medical School, University of East Anglia, Norwich, UK

**Keywords:** Exercise, Meta-analysis, Quality of life, Strength, Haematopoietic stem cell transplantation

## Abstract

**Purpose:**

To evaluate the effects of physical exercise on key clinical outcomes (such as quality of life (QoL), aerobic capacity, fatigue, and muscle strength) in patients undergoing haematopoietic stem cell transplantation.

**Methods:**

PRISMA systematic review and meta-analysis from inception to April 2025. PubMed, Embase, Cochrane Library, Web of Science and Scopus databases were searched for eligible studies. Methodological quality and risk of bias were evaluated with the Pedro scale and the Risk-Of-Bias Tool for randomized trials (ROB2.0) respectively.

**Results:**

An initial search retrieved 5217 articles. Finally, 20 studies were included in the qualitative synthesis and 7 in the meta-analysis. Exercise showed a small, non-significant effect on QoL (SMD = 0.23; *p* = 0.072), and a significant moderate effect on functional capacity (SMD = 0.43; *p* = 0.002). No significant effects were found on fatigue (SMD = − 0.05; *p* = 0.819) or handgrip strength (SMD = 0.21; *p* = 0.201), although a large and significant effect was observed for lower limb strength (SMD = 1.48; *p* < 0.001). Meta-regression analyses indicated that longer intervention duration was significantly associated with greater improvements in fatigue.

**Conclusions:**

Physical exercise improves functional capacity and lower limb strength in transplant patients, while effects on quality of life and fatigue are less consistent. Longer interventions are associated with greater improvements in fatigue. These results support the implementation of prolonged exercise programmes to optimize post-transplant recovery.

**Supplementary Information:**

The online version contains supplementary material available at 10.1007/s00520-025-10194-5.

## Introduction

Haematopoietic stem cell transplantation (HSCT) is a potentially curative treatment for many hematologic malignancies. Nevertheless, the procedure is associated with several adverse effects, including asthenia, loss of muscle mass and strength, functional decline, and reduced quality of life [1]. These effects may persist for months, negatively impacting both clinical recovery and the patient’s return to usual activity. The impact of HSCT on patients’ physical and psychological well-being underscores the need for complementary therapeutic strategies that promote faster, person-centred functional recovery [2].

In this context, physical activity has proven to be a safe and potentially beneficial intervention [3, 4]. Several studies have reported improvements in exercise tolerance, functional capacity, muscle strength, and perceived quality of life, while also alleviating symptoms such as cancer-related fatigue [5]. Therefore, international oncology care guidelines are increasingly incorporating physical exercise as a standard recommendation within comprehensive care programmes, with growing evidence suggesting that its implementation may reduce hospital stay duration, complication rates, and improve post-transplant functional outcomes. However, the heterogeneity in study designs, exercise modalities, and timing of interventions limits the generalisability of results and the development of robust clinical recommendations [6–8].

In the HSCT setting—where patients undergo long periods of isolation, immunosuppression, and physical deconditioning—exercise is positioned not only as a rehabilitative tool, but also as a strategy for preserving functionality, reducing the psychosocial impact of treatment, and enhancing overall health perception [9, 10]. Tailoring exercise programmes to the clinical status, transplant phase, and baseline functional capacity of each patient is essential to maximise benefits and ensure safety. Furthermore, a multidisciplinary approach and the integration of exercise into patient care can facilitate faster recovery and a more effective transition to active living following hospital discharge [11, 12].

Therefore, it is essential to critically and rigorously synthesise the current evidence in order to guide clinical practice and provide clear recommendations for healthcare professionals. This systematic review and meta-analysis aims to evaluate the effects of physical exercise on key clinical outcomes (such as quality of life, aerobic capacity, fatigue, and muscle strength) in patients undergoing HSCT, while also analysing the influence of variables such as intervention duration, frequency, and type. The findings of this work will help strengthen the role of physical exercise as a core component of supportive cancer care, with a focus on functional recovery, well-being, and patient autonomy.

## Material and methods

### Data sources and search strategy

This systematic review and meta-analysis was conducted in accordance with the PRISMA (Preferred Reporting Items fir Systematic Reviews and Meta-Analyses) guidelines [13], and it is registered in the International Prospective Register of Systematic Reviews (PROSPERO; registration number CRD420251030849. We searched Pubmed, Embase, Cochrane Library, Scopus and Web of Science (WOS) inception April 2025. Supplementary material 1 provides the search strategies, tailored for each database.

### Eligibility criteria

The inclusion criteria and description of studies in this review followed the PICOS (Population. Intervention, Comparison, Outcomes and Study design) framework for reviews [14].

#### Population

People undergoing HSCT participating in physical exercise programmes.

#### Intervention

Physical exercise programmes in any of their forms.

#### Comparison

The comparison groups received usual care or no treatment.

#### Outcomes

Quality of life, oxygen consumption, fatigue and strength.

#### Study design

Randomized controlled trials (RCTs), controlled trials or intervention studies were included.

### Study selection

Two independent reviewers (CMS and EJFR) carried out the search using an identical methodology. Any disagreements were addressed through consensus, involving a third reviewer (JLSG) when necessary. Furthermore, the reference of the original studies was examined manually, and the authors were contacted for further information if required.

### Data extraction

Data presented in the results were extracted using a protocol to ensure retrieval of the most relevant information for each study. For the report of the qualitative results, the type of study, haematological disease, type of transplant, age, groups, type of intervention, training volume (weeks, session frequency, and duration), measurement variables, and outcomes were extracted.

### Risk of Bias and the assessment of methodological quality of studies

A revised version of the Risk of Bias tool for randomised clinical trials (RoB2) [15] was employed to evaluate potential bias in the included trials. This instrument comprises five domains in which bias might be introduced and these were established based on empirical findings and theoretical rationale.

The methodological quality of the studies was assessed using the PEDro scale [16], which evaluates both internal and external validity based on 11 criteria and was overall score served as an indicator of its methodological quality: 9–10 = excellent; 6–8 = good; 4–5 = fair; and 0–3 = poor [17].

### Data synthesis and analysis

The extracted data were summarized in tables and forest plots. For each study, the mean difference (MD) between pre- and post-intervention was calculated separately for the intervention and control groups. These pre–post changes were then compared between groups to compute the standardized mean difference (SMD) and corresponding 95% confidence interval (CI), using Hedges’ g correction to account for small sample bias.

The overall effect size was estimated using a random-effects model, with restricted maximum likelihood (REML) to estimate between-study variance (τ^2^). The Knapp–Hartung adjustment was applied when appropriate to yield more robust confidence intervals for the pooled effect size. Heterogeneity was assessed using Cochran’s Q test (with a significance level of *p* < 0.10) and I^2^ statistics, interpreted as low (> 25%), moderate (> 50%), or high (> 75%) heterogeneity [18].

When heterogeneity was detected (I^2^ > 25% or significant Q test), an influence analysis was performed to identify potential outliers or influential studies, using Baujat plots, leave-one-out diagnostics, and influence statistics (standardized residuals, Cook’s distance, and covariance ratio) [19, 20]. Identified outliers or influential cases were carefully examined and excluded in sensitivity analyses when justified.

Subgroup analyses were conducted to explore potential sources of heterogeneity by grouping studies according to post-transplantation period (acute, subacute, or discharge), type of transplant (e.g. HSCT or solid organ) and exercise type (e.g., resistance, aerobic, or combined). These analyses aimed to determine whether these factors influenced the overall effect estimates. Subgroup analyses were only conducted for outcome measures in which each subgroup included at least two studies.

Meta-regression analyses were performed to examine the effect of continuous moderators on the pooled SMD, including participant characteristics such as mean age, and intervention characteristics such as duration (minutes), frequency (sessions/week), and length (weeks) of the exercise programme. For each moderator, the meta-regression coefficient, 95% CI, and *p*-value were reported.

Publication bias was assessed via contour-enhanced funnel plots for outcomes with ≥ 5 studies. Asymmetry was interpreted as potential evidence of small-study effects or publication bias.

All analyses were conducted using R software, specifically the metafor and meta packages, following the guidelines proposed by Harrer et al. Meta-analytical results were interpreted considering the magnitude of the effect (SMD: small = 0.2–0.5, moderate = 0.5–0.8, large > 0.8) [21], the heterogeneity, and the risk of bias.

## Results

### Search results and study selection

**Fig. 1:**
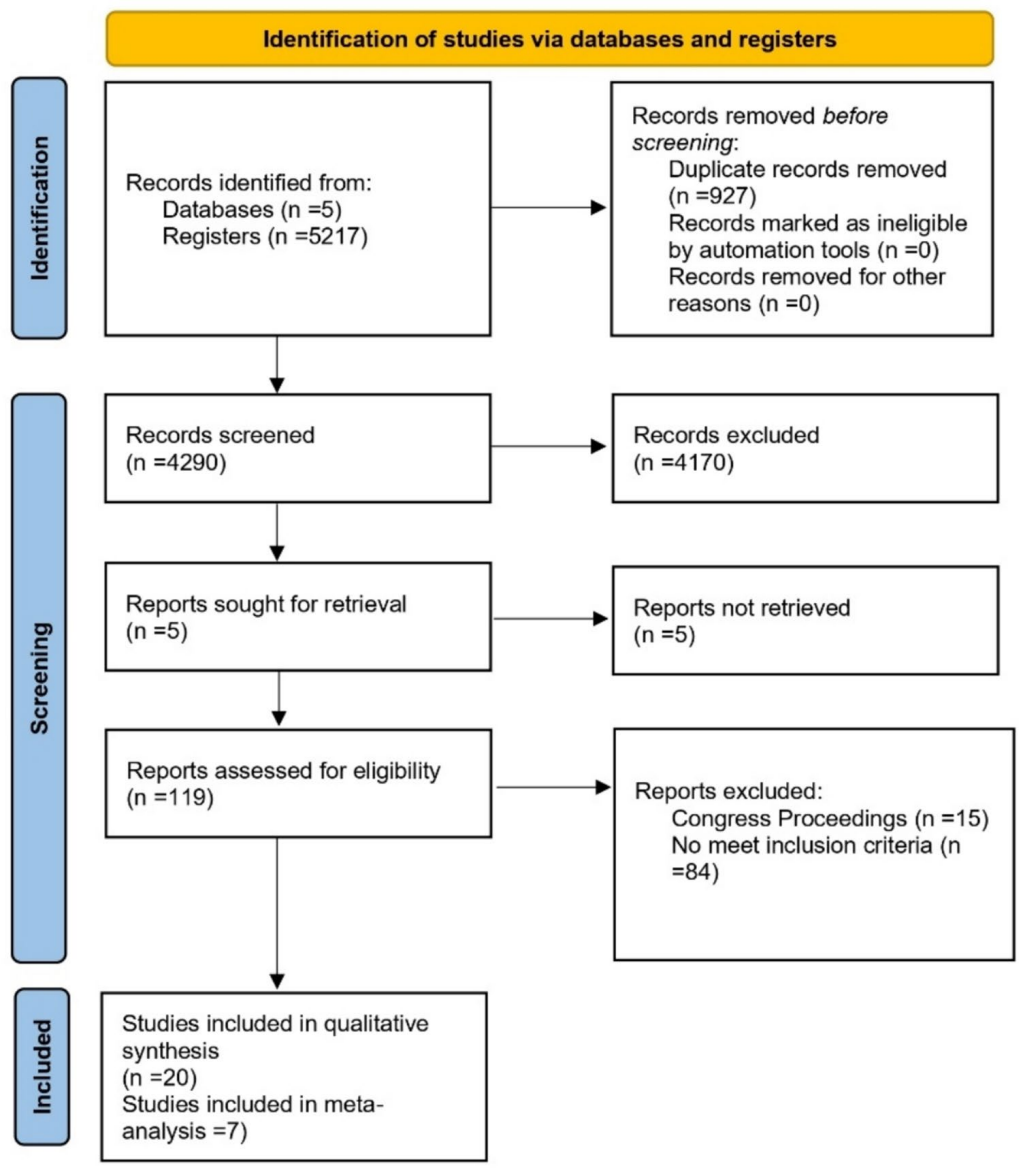
Flow diagram

The search found 5217 records, of which 927 were duplicates and 4290 were screened by title and abstract. We found 120 studies that were potentially relevant and excluded 104 after full review. Finally, 20 studies met the eligibility criteria and were included in the qualitative analysis, and 7 were included in the quantitative analysis. The entire screening process is shown in the PRISMA flow diagram in Fig. 1.

### Study characteristics

The 20 included studies represented 1697 patients (1123 in the intervention group and 574 in the control group). Three pilot randomized controlled trial [22–24], 8 randomized controlled trials [25–32], 4 non-randomized controlled trials [33–36] and 5 single arm studies [37–41] were included.

The average duration of the intervention was 9.52 weeks with a frequency of 5 days per week. Regarding the type of physical exercise used, 11 studies carried out their intervention with a combination of aerobic exercise and resistance training [22, 23, 25, 26, 29–31, 34, 35, 39, 40], 3 with aerobic exercise [27, 28, 33], 3 with high intensity interval training [32, 36, 37] and 3 studies with resistance training [24, 38, 41]. The other characteristics of the included studies are detailed in Table 1.

**Table 1:** Characteristics of studies included

Study	Study design	Population (disease)	Type of transplantation	Age (years)	Groups (*n*)	Type of Intervention	Training Volume			Outcomes	Results
							**Weeks**	**Frequency (sessions/week)**	**Session duration (minutes)**		
Coleman et al. 2003	Pilot RCT	Multiple myeloma	Autologous HSCT	55	Intervention group (14)	Aerobic and resistance training	32 weeks	7 days a week	Variably, the programme is home based	Aerobic capacity (Modified Balke protocol) Strength (1RM) Fatigue (POMS) Sleep quality (Actigraph) Anthropometric measurements (plethysmography “Bod Pod”)	Positive effect of exercise on leaning body weight
					Control group (10)	Usual care group	32 weeks	3 days a week	20 min		
Potiaumpai et al. 2021	RCT	Hematopoietic cancer	Autologous or allogeneic HSCT	59.26	Intervention group (19)	Aerobic training	2 weeks	7 days a week	The walking programme gradually increased in duration from 5 to 30 min	Physical function: - Aerobic capacity (6MWT) - Dynamic balance (Timed Up and Go) - Activities of Daily Living physical function (Physical performance test) HSCT-specific health-related-QOL (FACT-BMT)	HSCT-specific QOL showed moderate to large effect sizes and the suggested minimally important difference (MID) after the walking programme. No significant differences between groups in physical function status, however the WALK group exhibited significant increases in aerobic capacity after the intervention
				58.19	Control group (16)	Usual care group	2 weeks	N/R	N/R		
Van Haren et al. 2018	NRCT	Hematopoietic cancer	Autologous or allogeneic HSCT	58.36	Intervention group (14)	Aerobic and resistance training	4–6 weeks	2 days a week	90 min	Perceived QOL (SF-36) Fatigue (Checklist Individual Strength) VO2 peak (Astrand Test) Muscle strength (Handgrip dynamometer JAMAR)	Exercise program was moderately feasible, safe, well tolerated, and perceived as valuable The programme might be effective in increasing physical functioning, QOL, and fatigue
				48.93	Control group (15)	Usual care group	4–6 weeks	N/R	N/R		
Jacobsen et al. 2014	RCT	Hematopoietic cancer	Autologous or allogeneic HSCT	58	Exercise training (180)	Walking	26 weeks	3–5 days a week	20–30 min	QOL (SF-36) Distress (Cancer and Treatment Distress) Sleep quality (Pittsburgh Sleep Quality Index) Nausea (2 items formatted like the SF36 Bodily Pain subscale)	No benefit from including a single brief instruction in exercise or stress management for all HSCT recipients to reduce distress or improve physical or mental functioning during transplantation and recovery
				58	Stress management training (178)	Abdominal breathing, muscle relaxation and coping self-statements	26 weeks	3–5 days a week	20–30 min		
				57	Combination of exercise and stress management training (178)	Walking and abdominal breathing, muscle relaxation and coping self-statements	26 weeks	3–5 days a week	20–30 min		
				55	Usual care (175)	Usual care group	26 weeks	3–5 days a week	20–30 min		
McCourt et al. 2023	Pilot RCT	Multiple myeloma	Autologous HSCT	59.3	Intervention group (27)	Aerobic and resistance training	12 weeks	3 days a week	Minimum 30 min	Fatigue (FACIT-F) Aerobic capacity (6MWT) Strength (hand grip and sit stand test) Sedentary time (IPAQ)	Feasibility of the pilot trial in relation to recruitment rate, attrition, and acceptability of the intervention. Secondary outcomes were limited
				61.3	Control group (23)	Usual care group	12 weeks	N/R	N/R		
Dimeo et al. 1999	NRCT	Solid tumors or lymphoma	Autologous HSCT	40	Intervention group (27)	Aerobic training	Undetermined	7 days a week	30 min	Psychological status ((POMS) and the Symptom Checklist (SCL-90- R))	Aerobic training reduces treatment-related fatigue and improves the psychological state of cancer patients receiving HSCT
				40	Control group (32)	Usual care group	Undetermined	N/R	N/R		
Schuler et al. 2016	Single-arm pilot study	Hematopoietic cancer	Allogeneic HSCT	64.5	Intervention group (16)	Aerobic and resistance training	4 weeks	6 days a week	15–30 min	Strength (ISOMED) Aerobic capacity (6-MWT) QOL (EORTC-QLQ-FA13) Depression and anxiety (HADS)	15% adherence for participants that performed the strengthening exercises with platelets of 10,000 to 19,000/mm^3^ (*n* = 10), and 10% adherence with platelet levels of less than 10,000/mm^3^ (*n* = 7). No adverse symptoms occurred during the performance of the exercise programme. Nausea was found to be associated with reduced aerobic adherence (*r* = − 0.44; *P* = 0.05), whereas there was no significant relationship between exercise participation and other symptoms such as lightheadedness and fatigue
Artese et al. 2023	Single-arm pilot study	Hematopoietic cancer	Allogeneic HSCT	58.8	Intervention group (13)	Home-based, remotely monitored high-intensity interval trainig (HIIT) programme	15 weeks	3 days a week	30 min	VO2peak (CPET) Physical function (SPPB and 30-s sit-to-stand test) Aerobic capacity (6MWT)	69.2% completed the training sessions Median session adherence: 100% (IQR: 87–107) Interval adherence: 92% Participants met or exceeded target HR in 68.8 ± 34.8% of high-intensity intervals VO₂peak improved significantly (from 14.6 ± 3.1 to 17.9 ± 3.3 mL/kg/min; *p* < 0.001) Significant improvements in: 30-s sit-to-stand (from 13.8 ± 1.5 to 18.3 ± 3.3 reps) 6MWT (from 514.4 ± 43.2 m to 564.6 ± 19.3 m) SPPB chair stand scores Improvements exceeded minimal clinically important difference (MCID) thresholds established for other chronic disease populations
Wiskemann et al. 2014	RCT	Hematopoietic cancer	Allogeneic HSCT	47,6	Intervention group (52)	Aerobic and resistance training	1–4 weeks before hospital admission	Aerobic 3–5 days a week and resistance 2 days a week	20–40 min	Maximal voluntary isometric strength (knee-extensors, knee-flexors, hip-abductors, hip-flexors, elbow-extensors, elbow-flexors and shoulder-abductors) (hand-held dynamometer) Aerobic capacity (6MWT) Exhaustion (BORG Scale) Fatigue (MFI-20) QOL (EORTC QLQ-30) Distress (NCCN Distress Thermometer)	Intervention Group compared with control group: Significant different group developments for all measurements, except for the elbow flexors Changes in PROs No significant changes about PROs
				50	Control group (53)	Usual care	1–4 weeks before hospital admission	N/R	N/R		
Baumann et al. 2010	RCT	Hematopoietic cancer	Autologous or allogeneic HSCT	44,9	Intervention group (32)	Aerobic and resistance training	6 days before HSCT until 1 day before discharge	7 days a week	30–40 min: 10–20 min (aerobic) + 20 min (exercise programme of walking, stepping and stretching)	Endurance (W) Relative endurance (W/Kg) Strength extensor muscles of the thighs (Digimax) Inspiratory vital capacity (IVC) Forced vital capacity (FVC) (Flowscreen Version 6.00d) Blood count (Leucocytes, Plts, Hb) QOL (EORTC QLQ-C30)	Significant differences in the 3 endurance variables in favor of the intervention group (IG) Significant differences in muscle strength in favor of the IG At discharge, IG patients rated their quality of life higher on 12 out of 15 items compared to control group patients In hematological parameters, there were significant intragroup differences in both groups, but with slight results in favor of IG
				44,1	Control group (32)	Usual care (standard physiotherapy programme)	1 day after HSCT until 1 day before discharge	5 days a week	20 min		
Wiskemann et al. 2011	RCT	Hematopoietic cancer	Allogeneic HSCT	47,6	Intervention group (52)	Aerobic and resistance training	1–4 weeks before hospital admission	Aerobic 3–5 days a week and resistance 2 days a week	20–40 min	Fatigue (MFI) Psychological status (POMS) QOL (EORTC QLQ-C30) Aerobic capacity (6MWT) Maximal isometric voluntary strength (Elbow flexors and extensors, shoulder abductors, hip abductors, and flexors, knee flexors and extensors bilaterally (hand-held Dynamometer) Depression and anxiety (HADS) Distress (NCCN Distress Thermometer)	The exercise group showed a significant improvement in fatigue versus the control group, which even increased, as well as significant improvements in physical fitness/functioning and global discomfort. Physical fitness was significantly correlated with symptoms/variables
				50	Control group (53)	Usual care and information	2 weeks before hospital admission	2–3 days a week	30 min		
Wood et al 2020	RCT	Hematopoietic cancer	Allogeneic HSCT	52	Intervention group (6)	Interval exercise training	11 weeks	3–4 days a week	30 min	VO2 peak (cycle ergometry + facemask NRB1 + K4b2 Cosmed, + 6MWT) Maximum heart rate (cycle ergometry + polar heart-rate strap + 6MWT + accelerometer Fitbit) QOL and Physical function (PRO-CTCAE) Global Health and Physical Function (PROMIS)	No significant differences in change scores for VO2peak or 6MWT between intervention and control group
				52	Control group (10)	Usual care	7 weeks	N/R	N/R		
Potiaumpai et al. 2024	Pilot RCT	Hematopoietic cancer	Autologous or allogeneic HSCT	57,30	Intervention group (36)	Home-based exercise, resistance training and strength	7 weeks	5–7 days a week	30–45 min	Physical Function: Aerobic capacity (6MWT), (30-s chair Stand), (TUG), (SPPB), (Berg balance scale), (Handgrip)	No significant differences in physical function assessments between autologous and allogeneic HSCT at any study time among exercise group. Clinical improvements in favor of autologous group at + 100 days post-HSCT for 6MWT and 30-s Chair Stands tests Significant intragroup improvements in the exercise group in all physical function variables Significant differences between exercise group and control group in the final assessment only in SPPB and 30-s Chair Stand tests. Clinical differences between groups in favor of the exercise group in physical function
				63,47	Control group (38)	Usual Care + Educational programme	N/R	N/R	N/R		
Santa Mina D et al. 2020	RCT	Hematopoietic cancer	Allogeneic HSCT	50,4	Intervention group (15)	Aerobic and resistance training	8 weeks	3 days a week	40–60 min	Recruitment and retention, adverse events, and adherence VO2 peak (CPET) Gas Exchange (TrueOne 2400 gas analyzer) Aerobic capacity (6 MWT + 30-s chair-stand test) Body mass index % Fat (TBF-300 A, Tanita) Grip strength (hand dynamometer) Upper extremity strength (elbow flexors and extensors (MicroFET2)) QOL (EORTC QLQ-C30) Anxiety (GAD7) Severity of depression (PHQ-9) Fatigue (FACT-F + MFI) Self-efficacy to perform exercise (ESES)	Adherence to resistance exercise was far superior to adherence to aerobic exercise at all three phases of the intervention in the exercise group Intragroup clinical improvements in FACT-F and 6 MWT, as well as in grip strength and quality of life, at various time points during the study for the exercise group
				48,4	Control group (15)	Usual care	8 weeks	N/R	N/R		
Kisch et al. 2020	Single-arm pilot study	Recipients of allogeneic stem cell transplantation (various diseases)	Allogeneic HSCT	55,5	Intervention group (67)	Resistance training	4 weeks before HSCT continuing during in-patient care (median 4 weeks) and after discharge (not supervised)	7 days a week	30–60 min	Fatigue (MFI-20 Scale) General fatigue subscale (GF)	Reduce fatigue and prepare patients for transplantation both physically and mentally Additional focus on exercise should be given to female, older and high-risk patients
Takahiro et al. 2015	Single-arm pilot study	Recipients of allogeneic stem cell transplantation (various diseases)	Allogeneic HSCT	58	Intervention group (35)	Stretching, resistance training	2 weeks before HSC and during the admission of the procedure	5 5 days a week	20**–**40 min	Aerobic capacity (6MWT) Strength (Handgrip dynamometer) Body composition measurements (muscle mass, body fat, lean body mass, bone mass, and body water volume) evaluated using bioelectrical impedance analysis (BIA)	The 6MWT, handgrip strength, and muscle mass in the upper extremities and trunk decreased, but that of the lower extremities remained unchanged (The reduction was smaller in the high frequency group). Exercise therapy may be effective in maintaining lower extremity muscle mass
Mawson et al. 2021	Single-arm pilot study	Multiple myeloma	Autologous HSCT	65	Intervention group (23)	Aerobic and resistance training	6 weeks	6 days a week	Not specified	Rates of recruitment, adherence and adverse events Aerobic capacity (6MWT) Test and patient reported outcome measures (PROMs). Experience of study and intervention	The 6MWT increased and improved the physical fitness and overall mental health and wellbeing prior to HSCT
Wood et al.2016	NRCT	Recipients of stem cell transplantation for various diseases	Autologous or allogeneic HSCT	60,5	Autologous transplant recipients (20)	Home-based interval training	6 weeks	3 days a week	30 min	VO2peak (cycle ergometry) Aerobic capacity (6MWT)	Improvement in cardiorespiratory capacity (VO2 peak and 6MWT), more marked in pre-allogeneic transplant patients
				52,5	Allogeneic transplant recipients (20)	Home-based interval training	6 weeks	3 days a week	30 min		
Coleman et al. 2008	RCT	Multiple myeloma	Autologous HSCT	55 years	Intervention group (58)	Aerobic and resistance training	5–12 weeks	N/R	N/R	Aerobic capacity (6MWT) Stem cell collection, numbers of RBC (red blood cells), platelet transfusions during the transplantation and time-to-recovery after transplantation (number of days before white blood cell recovery with absolute neutrophil count of 2.0 or higher)	The walk test measurements showed a trend toward better exercise performance by the exercise group. Loss of aerobic capacity during treatment was less in the exercise group. The exercise group had significantly fewer red blood cell transfusions and fewer attempts at stem cell collection. Recovery and treatment response were not significantly different between groups after transplant
				32–74 years	Control group (62)	Usual care, written exercise recommendations	5–12 weeks	N/R	20 min		
Vejby et al. 2021	NRCT	Recipients of autologous and allogeneic stem cell transplantation	Autologous or allogeneic HSCT	52	First phase of the study: Intervention group (21)	Aerobic and resistance training	Hospital stay and 14 days post-discharge	7 days a week	N/R	Aerobic capacity (6MWT) Fatigue and quality of life (FACT)	Increased physical activity, improved 6MWT and improved fatigue and quality of life
					First phase of the study: Control group (22)	Usual care and recommendations	Hospital stay and 14 days post-discharge	7 days a week	N/R		
				56	Second phase of the study: Intervention group (20)	Aerobic and resistance training	24 weeks	7 days a week	N/R		

*RCT*, Randomized Controlled Trial; *NRCT*, Non-Randomized Controlled Trial; *HSCT*, Hematopoietic Stem Cell Transplant; ↑: Improvement;↓: Deterioration; ↔ : No changes; *BDI-FS*, The Beck Depression Inventory—Fast Screen; *BDI-II*, The Beck Depression Inventory; *BFI*, The Brief Fatigue Inventory; *CES‐D*, Centre for epidemiologic studies depression scale; *CG*, Control Group; *IG*, Intervention Group; *DASS-21*, Depression Anxiety Stress Scales-21; *EG*, Experimental Group; *EORTC‐QLQ‐C30*, European Organisation for Research and Treatment of Cancer Quality of Life Questionnaire – fatigue symptom scale; *EORTC‐QLQ‐C30‐GHS*, European organization for research and treatment of cancer quality of life—Global health status; *EORTC-QLQ-FA13*, EORTC Quality of Life Module Measuring Cancer Related Fatigue; *FACIT-F*, The Functional Assessment of Chronic Illness Therapy—Fatigue; *FACT-Anemia*, The anemia subscale of FACT-Cog; *FACT-Cog-QOL*, The QOL subscale of FACT-Cog; *FACT-BMT*, The Functional Assessment of Cancer Therapy-Bone Marrow Transplantations; *MFI-20*, Multidimensional Fatigue Inventory; *HADS*, Hospital anxiety and depression scale; *HADS-A*, Hospital anxiety and depression scale-Anxiety scale; *MIA-A*, Metamemory in Adulthood-Anxiety scale; *N/R*, Not Reported. *FACT-Cog*, Cancer Treatment–Cognitive Function; *POMS*, The profile of mood status; *BAI*, Beck Anxiety Inventory; *QOL-CS*, QOL—Cancer Survivors; *SF-36*, The 36-item Short Form Health Survey; *SF-12*, The 12-Item Short Form Health Survey; *STAI*, The State-Trait Anxiety Inventory; *STAI-S*, Spielberger State-Trait Anxiety Inventory-State Subscale; *WHOQOL-BREF*, The World Health Organization–abridged version; *6MWT*, 6-min walk test; *PROMs*, Patient Reported Outcome Measures; *RBC*, Red Blood Cells; *RM*, One-rep max; *MID*, Minimally important difference; *VO2max*, Maximal Oxygen Consumption; *IPAQ*, Physical Activity Questionnaire; *SCL-90-R*, The Symptom Checklist; *CPET*, Cardiopulmonary Exercise Test; *SPPB*, Short Physical Performance Battery; *NCCN*, National Comprehensive Cancer Network; *W*, Vatio; *KG*, Kilograms; *IVC*, Inspiratory Vital Capacity; *FVC*, Forced Vital Capacity; *PRO-TCAE*, Patient Reported Outcomes in Cancer Clinical Trials; *TUG*, Timed Up and Go; *GF*, General Fatigue Subscale; *BIA*, Bioelectrical Impedance Analysis; *CIS*, Checklist Individual Strength; *PHQ-9*, Patient Health Questionnaire; *GAD7*, Generalized Anxiety Disorder; *ESES*, Exercise Self-Efficacy Scale

### Meta-analyzed

#### Effects of exercise on quality of life

**Fig. 2:**
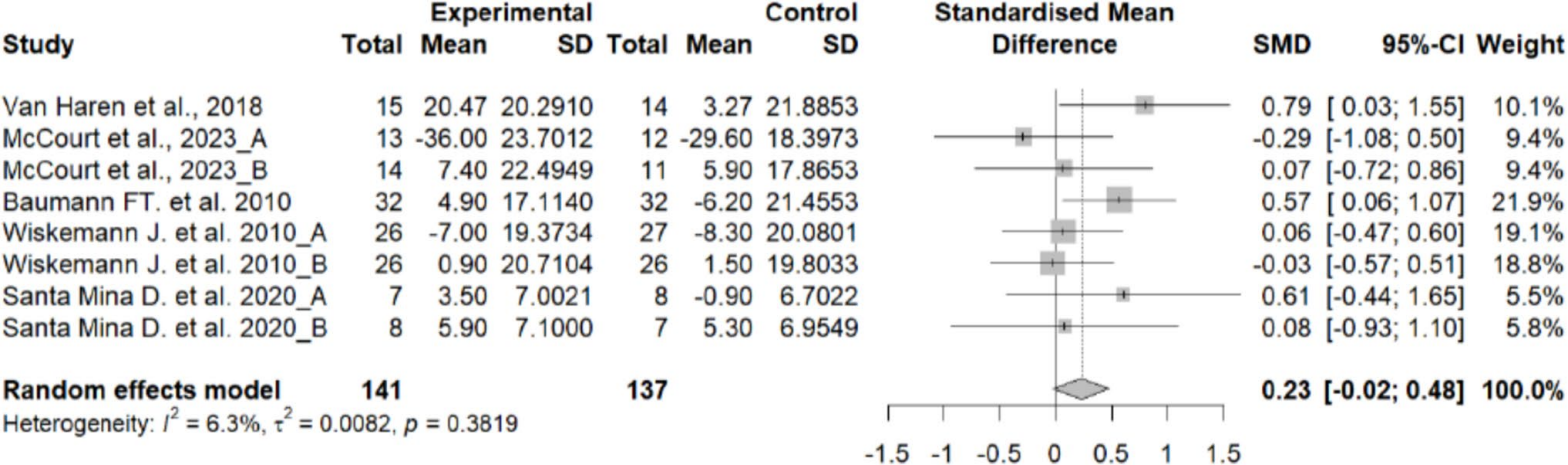
Forest plot of the effects of exercise on quality of life

The QOL was higher in the experimental group compared to the control group, showing a small effect size (SMD = 0.23; 95% CI: − 0.02 to 0.47; *n* = 278; Z = 1.80; *p* = 0.072), which approached statistical significance. Heterogeneity was low (I^2^ = 6.3%; *p* = 0.381) (Fig. 2). The funnel plot showed some asymmetry, suggesting a potential risk of publication bias (Supplementary material 1).

Subgroup analyses were conducted according to post-transplantation period and type of transplant and no significant differences either according to the post-transplantation period (acute, subacute, or discharge) (*p* = 0.916) (Supplementary material 2) or by the type of transplant (hematopoietic stem cell vs. solid organ) (*p* = 0.057) (Supplementary material 3), indicating that these factors did not significantly influence the effect on quality of life.

### Effects of exercise on oxygen consumption

**Fig. 3:**
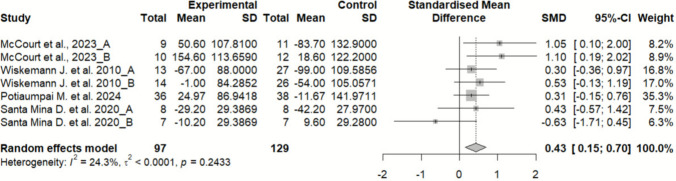
Forest plot of the effects of exercise on oxygen consumption

The meter walked in the 6MWT was significantly lower in the control group compared to the experimental group, showing a medium effect size (SMD = 0.43; 95% CI: 0.15 to 0.7; *n* = 226; Z = 3.06; *p* = 0.002). Heterogeneity was moderate (I^2^ = 24.3%; *p* = 0.243) (Fig. 3). The funnel plot showed some asymmetry, suggesting a potential risk of publication bias (Supplementary material 4).

In the subgroup analysis, no significant differences were found according to the post-transplant period (acute, subacute or at discharge) (*p* = 0.916) (Supplementary material 5) or the type of transplant (hematopoietic stem cells versus solid organ) (*p* = 0.057) (Supplementary material 6,7), indicating that these factors did not significantly influence the effect on quality of life.

#### Effects of exercise on fatigue

**Fig. 4:**
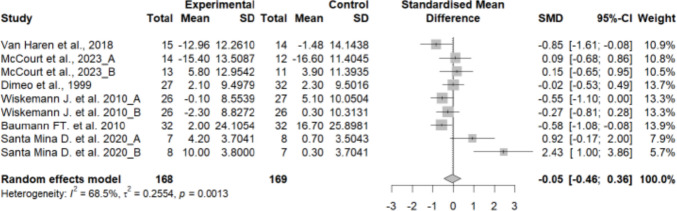
Forest plot of the effects of exercise on fatigue

Fatigue did not differ significantly between the experimental and control groups, showing a small effect size (SMD = − 0.05; 95% CI: − 0.46 to 0.36; *n* = 337; Z = 0.23; *p* = 0.819). Heterogeneity was high (I^2^ = 68.5%; *p* = 0.001) (Fig. 4). The funnel plot showed no clear asymmetry, indicating a low risk of publication bias (Supplementary material 8).

**Fig. 5:**
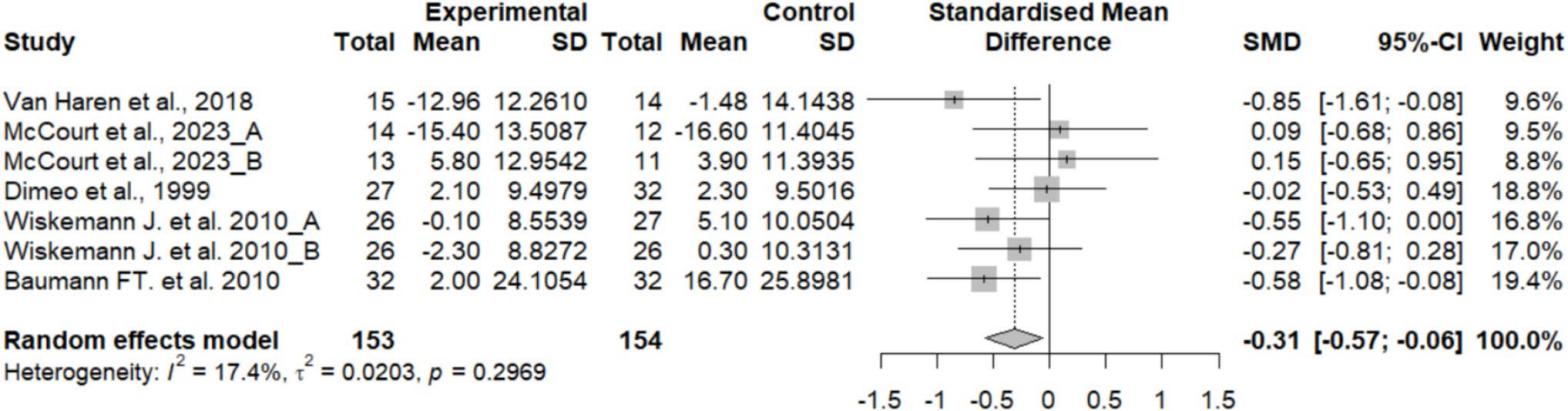
Forest plot of the effects of exercise on fatigue after sensitivity analysis excluding Santa Mina et al

Due to the high heterogeneity, a sensitivity analysis was conducted excluding the study by Santa Mina D. et al. 2020, which was identified as an outlier through influence analysis methods and outlier detection techniques, including Leave-One-Out analysis, Baujat plot, and standardized residuals assessment (Supplementary material 9, 10). The exclusion of this study changed the significance of the overall effect, showing that it changed from (SMD = − 0.05; 95% CI: − 0.46 to 0.36; *n* = 337; Z = 0.23; *p* = 0.819) to (SMD = − 0.31; 95% CI: − 0.57 to − 0.06; *n* = 307; Z = 2.43; *p* = 0.015), indicating that this had a considerable impact on the heterogeneity and overall results of the meta-analysis (Fig. 5).

Subgroup analyses could not be performed because several subgroups included only a single study.

#### Effects of exercise on strength

**Fig. 6:**
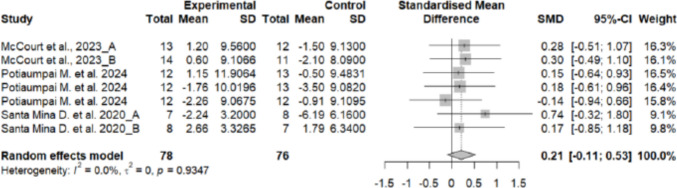
Forest plot of the effects of exercise on hand grip strength

Handgrip strength did not differ significantly between the experimental and control groups, showing a moderate effect size (SMD = 0.21; 95% CI: − 0.11 to 0.53; *n* = 154; Z = 1.28; *p* = 0.201). Heterogeneity was low (I^2^ = 0%; *p* = 0.934) (Fig. 6). The funnel plot showed no clear asymmetry, indicating a low risk of publication bias (Supplementary material 11).

No significant differences were found between subgroups based on the type of transplant (hematopoietic stem cell vs. solid organ) (*p* = 0.652) (Supplementary material 12) or the type of exercise (aerobic + resistance or resistance + cooldown) (*p* = 0.385) (Supplementary material 13), indicating that these factors did not significantly influence the effect on quality of life.

**Fig. 7:**
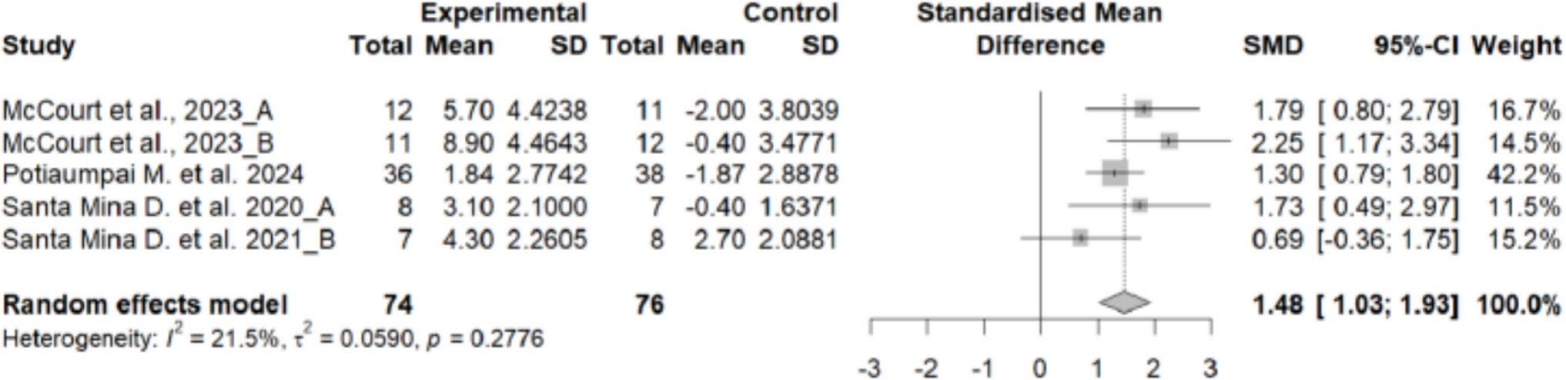
Forest plot of the effects of exercise on lower limb strength

The lower limb strength was significantly lower in the control group compared to the experimental group, showing a high effect size (SMD = 1.48; 95% CI: 1.03 to 1.93; *n* = 150; Z = 6.42; *p* < 0.001). Heterogeneity was low to moderate (I^2^ = 21.5%; *p* = 0.278) (Fig. 7). The funnel plot showed some asymmetry, suggesting a potential risk of publication bias (Supplementary material 14).

Subgroup analyses could not be performed because several subgroups included only a single study.

### Sensitivity analysis

A leave-one-out sensitivity analysis was conducted to assess the influence of individual studies on the overall effect for the studied outcomes (Supplementary material 15).

### Meta-regression analysis

In the meta-regression analyses conducted to explore potential moderators across different outcomes, five variables were tested: the mean age of the intervention group, the mean age of the control group, the duration of the intervention in weeks, the weekly frequency of the sessions, and the duration of each session. Among these, only the duration of the intervention showed a statistically significant association with the effect on fatigue.

Eight studies (k = 8) were included in this analysis. Residual heterogeneity remained moderate to high (tau^2^ = 0.2489, I^2^ = 64.96%), although the model explained 24.2% of the total heterogeneity (R^2^ = 24.2%). The test for residual heterogeneity (QE) was significant (*p* = 0.009), indicating that unexplained variability among the studies persisted. However, the test of moderators (QM) confirmed that the number of intervention weeks was a significant predictor of the effect on fatigue (*p* = 0.016). The positive coefficient (0.095) suggests that longer interventions are associated with greater improvements in fatigue (Supplementary material 16, 17).

### Risk of bias

**Fig. 8:**
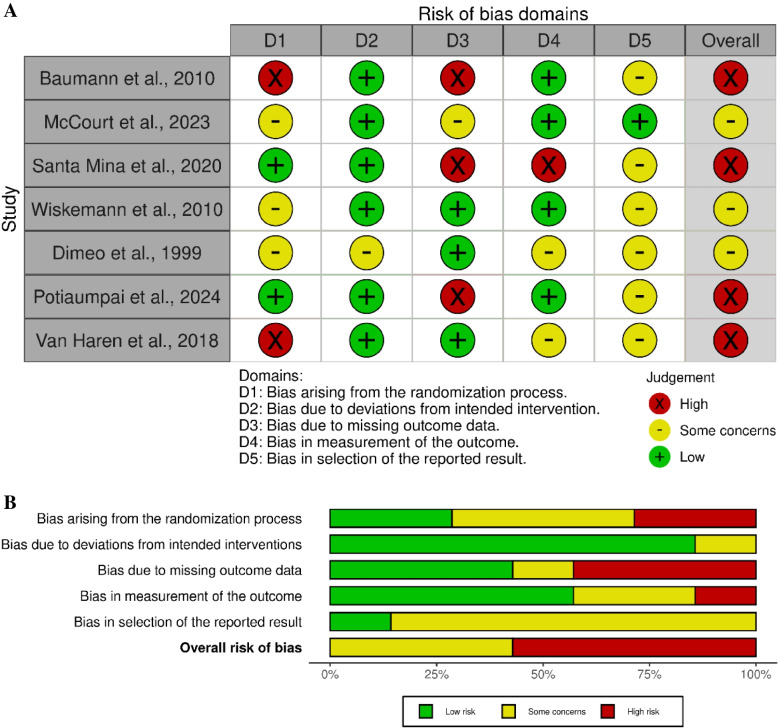
**A/B** Risk of bias of studies included

As assessed by RoB2, 57% (4/7) of the studies showed a high risk of bias and 43% (3/7) showed some concerns. The item with the highest risk of bias was “bias due to missing outcome data” in which 43% (3/7) of the studies showed a high risk of bias (Fig. 8A/B).

### Methodological quality

The methodological quality score ranged from 3 to 7 out of a maximum of 10 points (Table 2). The mean methodological quality score of the included studies was 5.4. 4 studies had “good” methodological quality; 2 had “fair” methodological quality and 1 study had “poor” methodological quality. The most frequent biases were related to therapist and patient blinding.

**Table 2:** Methodological score of randomized clinical trials using the Physiotherapy Evidence Database (PEDro) scale

Study	1	2	3	4	5	6	7	8	9	10	11	Total
Baumann FT. et al. 2010	Y	Y	N	N	N	N	N	Y	N	Y	Y	4/11
Dimeo et al., 1999	Y	Y	N	Y	N	N	N	Y	Y	Y	Y	6/11
McCourt et al., 2023	Y	Y	Y	Y	N	N	Y	N	Y	Y	Y	7/11
Potiaumpai M. et al. 2024	Y	Y	Y	Y	N	N	Y	N	Y	Y	Y	7/11
Santa Mina D. et al. 2020	Y	Y	Y	Y	N	N	N	N	Y	Y	Y	6/11
Wiskemann J. et al. 2010	Y	Y	N	Y	N	N	N	N	Y	Y	Y	5/11
Van Haren et al., 2018	Y	N	N	N	N	N	N	N	Y	Y	Y	3/11

Y = yes; N = no.

1: eligibility criteria; 2: random allocation of participants; 3: concealed allocation; 4: similarity between groups at baseline; 5: participant blinding; 6: therapist blinding; 7: assessor blinding; 8: dropout rate less than 15%; 9: intention-to-treat analysis; 10: between-group statistical comparisons; 11: point measures and variability data

## Discussion

This systematic review and meta-analysis provides an updated overview of the effects of physical exercise in patients undergoing HSCT, focusing on quality of life, oxygen consumption, fatigue, and muscle strength. It includes 20 studies in qualitative analysis and 7 in quantitative synthesis. The evidence confirms that exercise programmes are a safe and effective strategy for HSCT recipients, showing benefits in quality of life, oxygen consumption, and lower limb strength. However, improvements in fatigue and upper limb strength, while favorable, were not statistically significant. Although subgroup and meta-regression analyses did not reveal significant moderating effects, overall trends offer clinically relevant insights for exercise prescription after transplantation. Combined aerobic and resistance programmes appear most beneficial for enhancing functional capacity and strength across post-transplant phases. The consistent direction of effects across subgroups supports the safe and effective implementation of exercise from the acute to the discharge phase, regardless of transplant type. Moreover, longer and more frequent interventions were associated with greater gains in oxygen consumption and strength, suggesting that sustained, multimodal exercise may produce the most meaningful clinical benefits in this population.

The duration of physical exercise programmes is a fundamental of the positive effects found in different variables such as functional capacity, muscle strength, and fatigue. These improvements appear due to a combination of physiological mechanisms and behavioral aspects [42].

From a physiological point of view, sustained physical exercise improves cardiovascular and mitochondrial function and muscle oxidative capacity, which leads to better oxygen utilization and greater endurance. These adaptations trigger improvements in functional capacity or lower limb strength, commonly reported in the existing literature [43]. On the other hand, physical exercise modulates systemic inflammation and regulates immune function, improving low-level chronic inflammatory states and oxidative stress, which are common alterations in cancer patients undergoing treatment. The anti-inflammatory effect improves cancer-related fatigue and promotes proper overall metabolic function. Similarly, the neuroendocrine system benefits from regular physical exercise. Among the most obvious improvements are the normalization of hypothalamic–pituitary–adrenal axis activity, improved sleep quality, and regulation of circadian rhythms, leading to a secondary improvement in the patient’s quality of life and psychological well-being [44].

From a behavioral perspective, longer exercise programmes help patients gradually adapt to training and adopt healthier lifestyles. Furthermore, programmes lasting more than 12 weeks improve adherence and workload adequacy, allowing for gradual progression in exercise intensity and minimizing potential adverse effects [45].

The included exercise interventions varied considerably in type, intensity, duration, and frequency. The meta-analysis considered studies implementing combined aerobic and resistance training, isolated aerobic or resistance training, and high-intensity interval training. The beneficial effects of therapeutic exercise programmes appear to be closely linked to the modality, duration, frequency, and intensity of the training sessions. Otherwise, one strength of this meta-analysis is the homogeneity of the study population, exclusively composed of patients with hematologic malignancies treated with HSCT.

### Quality of life

Exercise interventions showed a trend toward improved quality of life (SMD = 0.23; *p* = 0.072), with low heterogeneity and no significant influence of transplant type or post-transplant phase. Although the pooled effect did not reach statistical significance, our findings are consistent with prior meta-analyses reporting small-to-moderate but clinically relevant benefits in HSCT populations. Persoon et al. [46] observed a moderate improvement in overall quality of life (SMD = 0.40; 95% CI 0.10–0.70), while Prins et al. [9] found similar effects (SMD = 0.27; 95% CI 0.05–0.49) in combined exercise–nutrition interventions. Likewise, Abo et al. reported a mean difference of + 3.38 points in the FACT-G questionnaire after exercise [47], and van Haren et al. [34] found a weighted mean difference of + 8.72 points at hospital discharge, reflecting measurable physical and psychological gains. In our meta-analysis, excluding the study by McCourt et al. [23] slightly increased the pooled effect to statistical significance (SMD = 0.28; *p* = 0.028), suggesting that exercise produces consistent improvements in HRQoL across studies. Taken together, these results support the role of structured exercise—particularly multicomponent aerobic-resistance programmes—in enhancing well-being, emotional stability, and overall life satisfaction in HSCT recipients.

### Oxygen consumption

Exercise significantly improved performance on the 6-Minute Walk Test (SMD = 0.43; *p* = 0.002), indicating a moderate effect size with low-to-moderate heterogeneity. These findings were consistent across sensitivity analyses and align with prior evidence showing that exercise enhances aerobic tolerance and functional capacity after transplantation. Liang et al. reported a similar effect (SMD = 0.45; 95% CI 0.25–0.65), equivalent to an average gain of ~ 40 m in 6MWT distance [12], while Morales Rodríguez et al. [48] found a + 36 m increase (*p* < 0.01). Likewise, Abo et al. [47] observed a + 29 m improvement, and Persoon et al. [46] reported a moderate effect on cardiorespiratory fitness (SMD = 0.53; 95% CI 0.20–0.86). The minimal subgroup variability found in our analysis suggests these benefits are consistent across transplant types, recovery phases, and exercise modalities. Overall, the evidence supports that exercise interventions effectively enhance oxygen uptake and walking endurance, promoting faster recovery and improved daily functioning after HSCT.

### Fatigue

No significant differences were found in the primary fatigue analysis (SMD = − 0.05; *p* = 0.819), with high heterogeneity. However, sensitivity analysis revealed that excluding Santa Mina et al. [29] notably altered the result, showing a significant reduction in fatigue (SMD = − 0.31; *p* = 0.015). The influence analysis confirmed that the study by Santa Mina et al. [29] had a substantial impact on the pooled estimate, markedly contributing to the observed heterogeneity and altering the overall direction of the effect. This finding indicates that the conclusions regarding fatigue should be interpreted with caution. This discrepancy may be attributed to methodological or intervention-related differences (e.g., intervention length, exercise intensity). Meta-regression identified intervention duration as a significant moderator (*p* = 0.016), indicating that longer interventions may be required to achieve clinically meaningful improvements in fatigue. Positive results regarding fatigue have been previously observed in other meta-analyses, showing moderate effects.

### Muscle strength

The analysis revealed differential effects depending on the muscle group assessed. Handgrip strength did not differ significantly between groups (SMD = 0.21; *p* = 0.201), whereas lower limb strength was significantly higher in the intervention group, with a large effect size (SMD = 1.48; *p* < 0.001). These findings suggest that exercise programmes in this population more effectively target large muscle groups in the lower extremities. This is consistent with previous literature, which has also reported improvements in lower but not upper limb strength following similar interventions [9, 12, 46, 48].

Overall, the results of this study expand and update the findings reported in previous meta-analyses. The study by Prins et al., [9] found significant improvements in physical exercise in the distance covered in the 6MWT (SMD = 0.41), lower limb muscle strength (SMD = 0.37), and global quality of life (SMD = 0.27), results that coincide with those presented in this study. Furthermore, Prins et al. [9] highlighted the safety and feasibility of exercise in HSCT recipients, our review strengthens these conclusions by demonstrating that extended interventions yield clinically meaningful gains in lower limb strength and fatigue reduction. Persoon et al. [46] reported similar benefits in cardiorespiratory fitness (SMD = 0.53), lower extremity muscle strength (SMD = 0.56) and fatigue (SMD = 0.53), consistent with our results. Similarly, the meta-analysis [47] showed an increase of 29 m in the 6MWT, an improvement in overall quality of life (MD = 3.38 points), and in fatigue (MD = 2.52 points), again coinciding with the results just presented. The meta-analysis [49] observed significant improvements in quality of life at the moment of discharge from the hospital (WMD = 8.72) and a decrease in fatigue (SMD = 0.53), partially coinciding with our results. On the other hand, our study reinforces the existing evidence in previous literature on the direct relationship between increased physical exercise intervention time and better results in the variables studied, an aspect that was not always evaluated in depth in previous studies.

In summary, this meta-analysis reinforces the benefits reported in previous studies and provides new insights. The duration of the intervention is postulated as a key factor in achieving significant improvements after training, and the benefits obtained are concentrated in aspects such as lower limb strength and functional capacity. Upper limb strength and cardiorespiratory function also benefited after training, but the results were less consistent. These new findings provide relevant information for the design of future physical exercise intervention programs.

### Limitations

This meta-analysis has several limitations that must be acknowledged. First, the number of studies included in the quantitative analyses was small (*n* = 7), limiting the generalizability of the findings and reducing statistical power to detect significant subgroup or moderator effects. Second, the methodological quality of the included studies was variable, with more than half showing a high risk of bias—primarily due to lack of blinding and incomplete outcome data. Third, there was considerable heterogeneity in the exercise protocols regarding type, intensity, duration, and frequency, which hinders direct comparison between studies and may have contributed to the variability observed in some outcomes.

### Clinical implications and future research

This meta-analysis supports structured physical exercise programs in routine care for patients undergoing HSCT. Improvements in functional capacity, quality of life, and lower limb strength indicate benefits for functional independence and post-hospital recovery. However, fatigue effects require confirmation through more rigorous, long-term randomized trials. Future research should refine intervention parameters (duration, frequency, intensity, and type) and develop guidelines tailored to transplant phase and clinical status.

Overall, exercise programs appear safe and effective to enhance quality of life, functional capacity, and muscle strength—especially in the lower limbs. Although fatigue outcomes were modest and methodologically sensitive, results favor incorporating exercise into post-transplant rehabilitation.

Intervention duration was a key moderator, particularly for fatigue, highlighting the need for sustained programs. In summary, this review underscores physical exercise as a valuable complementary therapy in HSCT care, with important clinical and research implications.

## Conclusions

Physical exercise in patients undergoing HSCT seems to improve functional capacity and lower limb strength. Quality of life showed a favorable but non-significant trend, and fatigue did not significantly improve unless an outlier study was excluded, highlighting the importance of intervention duration. No significant effects were observed on handgrip strength. Overall, these findings support the integration of prolonged exercise programs as part of post-transplant care to enhance functional recovery and alleviate fatigue.

## Supplementary Information

Below is the link to the electronic supplementary material.

ESM 1 (DOCX 9.50 KB)

ESM 2 (DOCX 2.45 MB)

## Data Availability

No datasets were generated or analysed during the current study.
