# Supplementary material for: Effects of physical exercise in patients undergoing haematopoietic stem cell transplantation: systematic review and meta-analysis
Source: Support Care Cancer. 2025 Dec 2;33(12):1160. doi: 10.1007/s00520-025-10194-5 (PMC12672829; doi:10.1007/s00520-025-10194-5)
Supplement: Supplementary file 1 — (DOCX 9.50 KB) [file 520_2025_10194_MOESM1_ESM.docx]

| Supplementary Material 1: Database formulas during literature search (09/04/2025) |
| --- |
| **PubMed Search Formula: 2502**  (physical activity OR exercise OR aerobic exercise OR acute exercise OR sports OR physical fitness OR exercise therapy OR resistance training OR muscle training OR exercise training OR physical exercise) AND (Allogeneic stem cell transplant OR Allo-transplant OR Stem cell transplant OR haematopoietic stem cell transplantation) |
| **Cochrane Library Search Formula: 494**  (physical activity OR exercise OR aerobic exercise OR acute exercise OR sports OR physical fitness OR exercise therapy OR resistance training OR muscle training OR exercise training OR physical exercise) en Título Resumen Palabra clave AND (Allogeneic stem cell transplant OR Allo-transplant OR Stem cell transplant OR haematopoietic stem cell transplantation) in Title Abstract Keyword |
| **SCOPUS Search Formula: 1001**  TITLE-ABS-KEY (physical activity OR exercise OR aerobic exercise OR acute exercise OR sports OR physical fitness OR exercise therapy OR resistance training OR muscle training OR exercise training OR physical exercise) AND (Allogeneic stem cell transplant OR Allo-transplant OR Stem cell transplant OR haematopoietic stem cell transplantation) |
| **WOS Search Formula: 182**  (physical activity OR exercise OR aerobic exercise OR acute exercise OR sports OR physical fitness OR exercise therapy OR resistance training OR muscle training OR exercise training OR physical exercise) en Título Resumen Palabra clave AND (Allogeneic stem cell transplant OR Allo-transplant OR Stem cell transplant OR haematopoietic stem cell transplantation) (All Fields) |

**Embase Search Formula: 459**

(physical activity OR exercise OR aerobic exercise OR acute exercise OR sports OR physical fitness OR exercise therapy OR resistance training OR muscle training OR exercise training OR physical exercise) AND (Allogeneic stem cell transplant OR Allo-transplant OR Stem cell transplant OR haematopoietic stem cell transplantation)
