# Supplementary material for: Effects of physical exercise in patients undergoing haematopoietic stem cell transplantation: systematic review and meta-analysis
Source: Support Care Cancer. 2025 Dec 2;33(12):1160. doi: 10.1007/s00520-025-10194-5 (PMC12672829; doi:10.1007/s00520-025-10194-5)

Supplementary material

**Figure S1 – Funnel plot QOL**


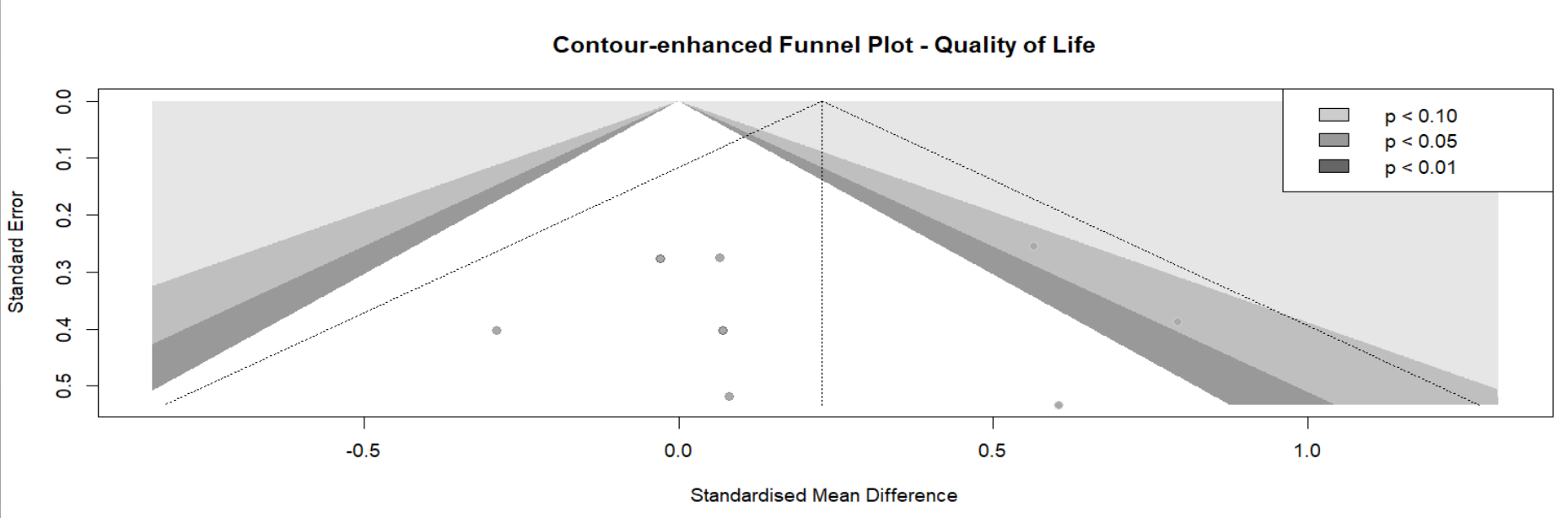


**Figure S2 - Subgroup analysis QOL: period**


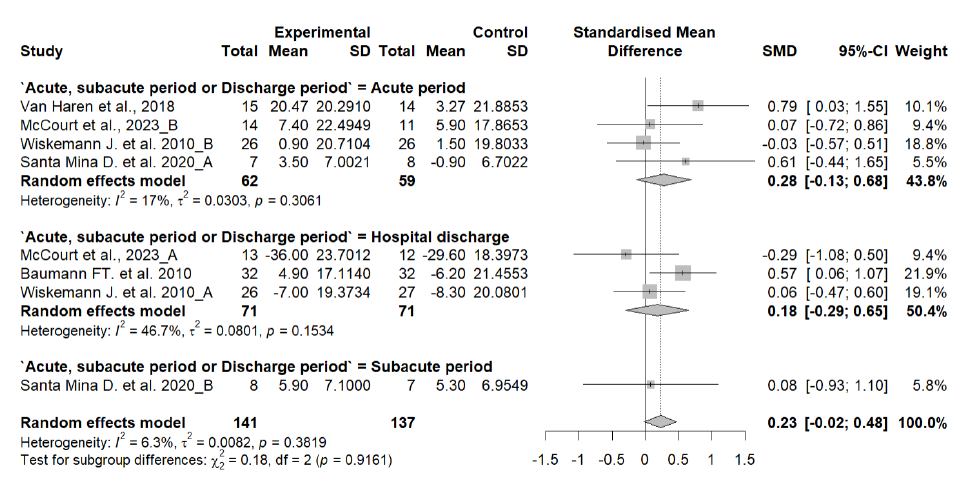


**Figure S3 - Subgroup analysis QOL: type of transplant**


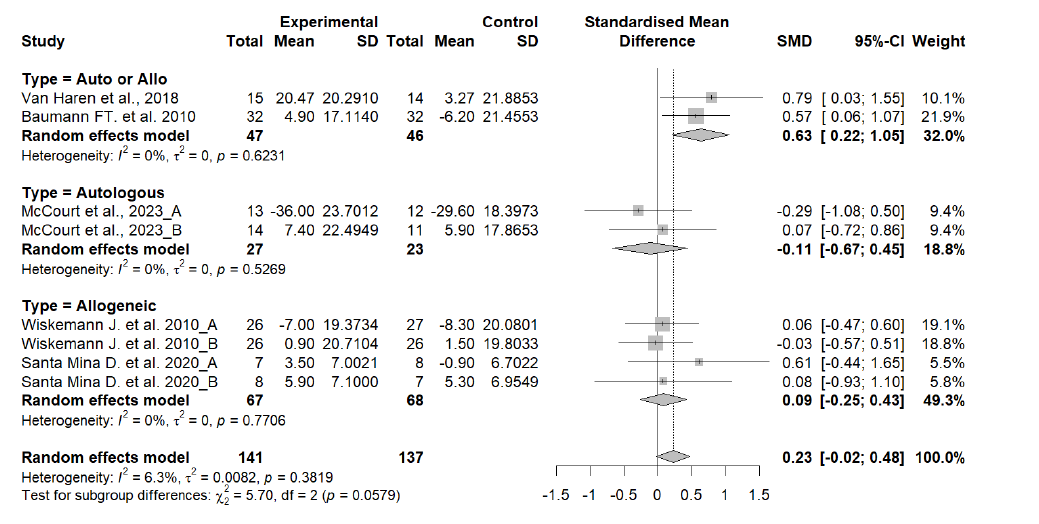


**Figure S4 – Funnel plot oxygen consumption**


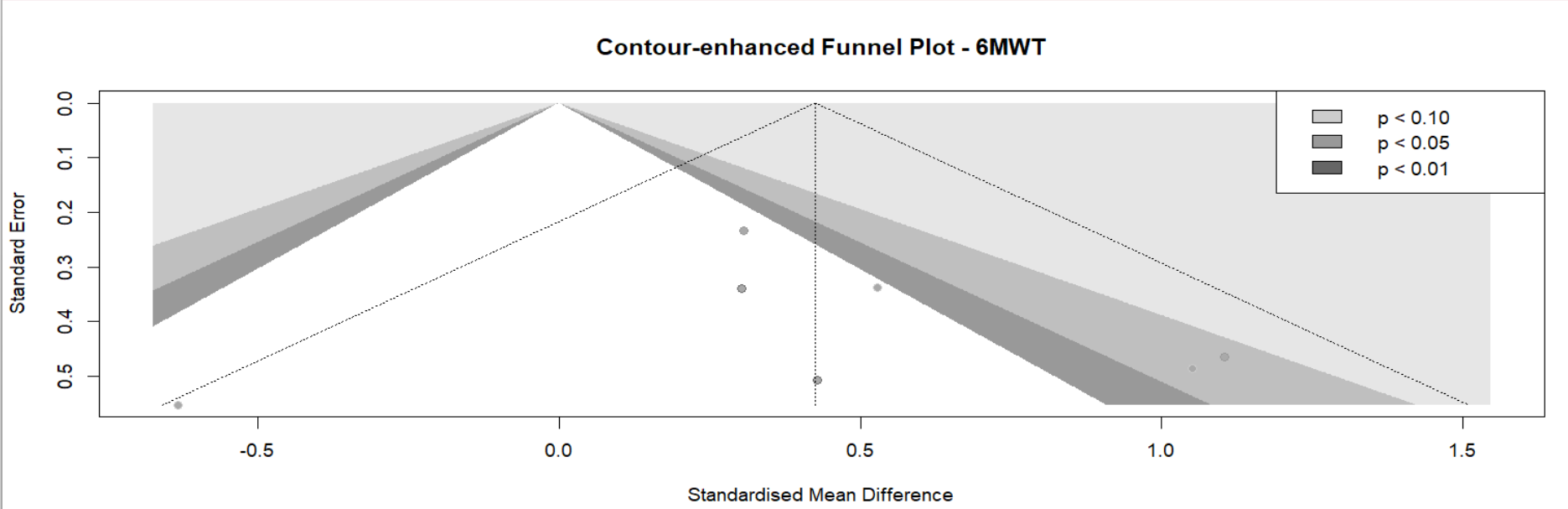


**Figure S5 - Subgroup analysis oxygen consumption: period**


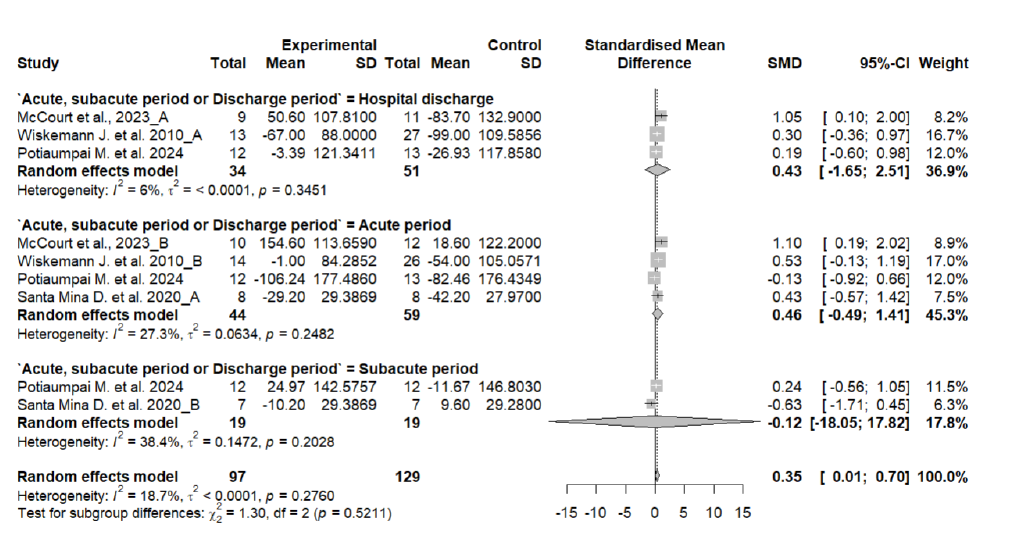


**Figure S6 - Subgroup analysis oxygen consumption: period**


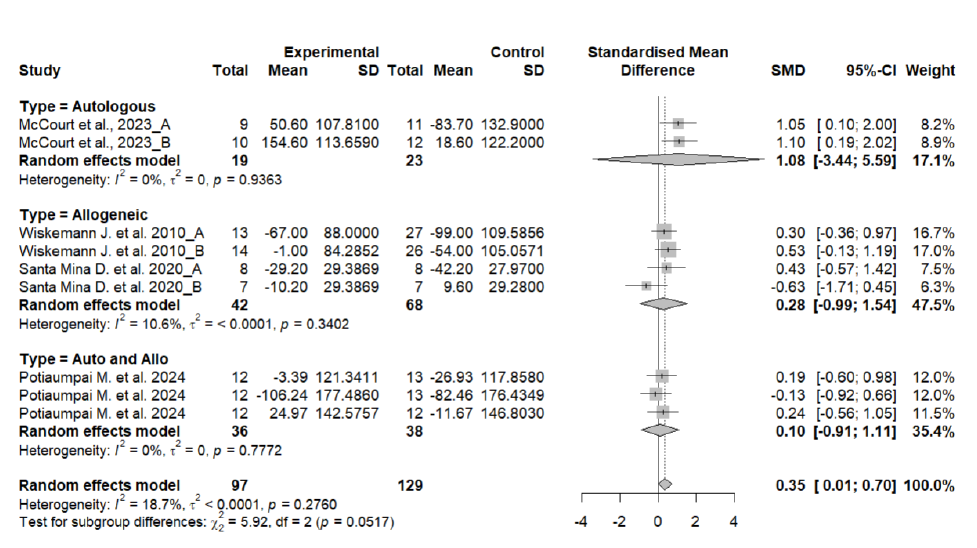


**Figure S7 - Subgroup analysis oxygen consumption: type of exercise**


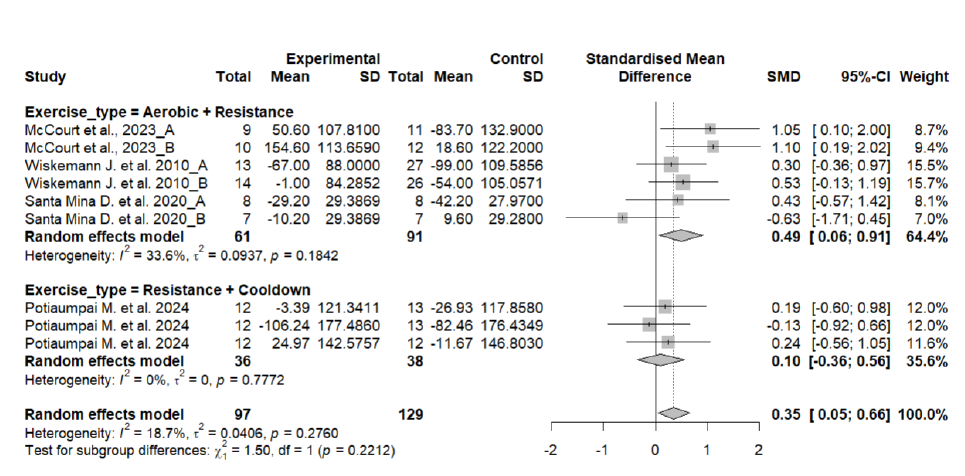


**Figure S8 – Funnel plot fatigue**


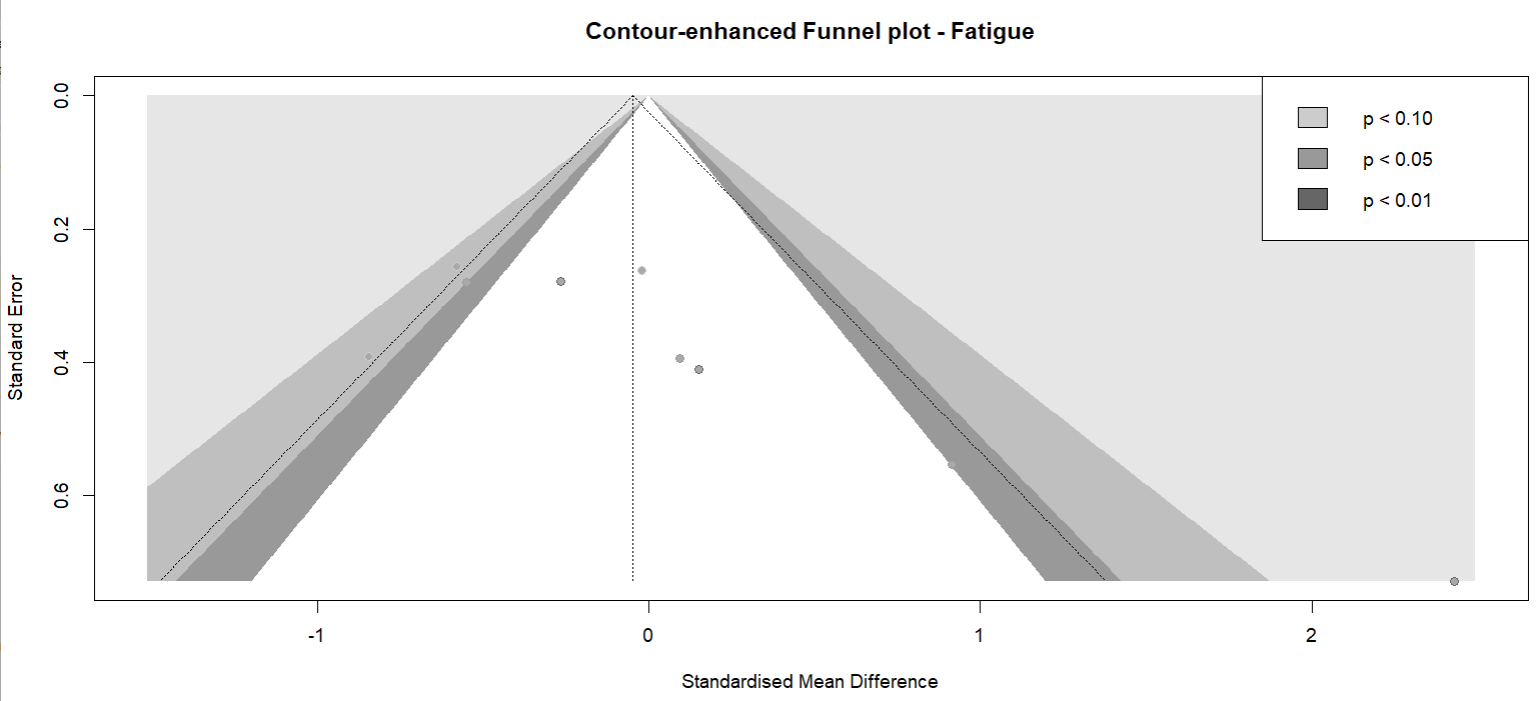


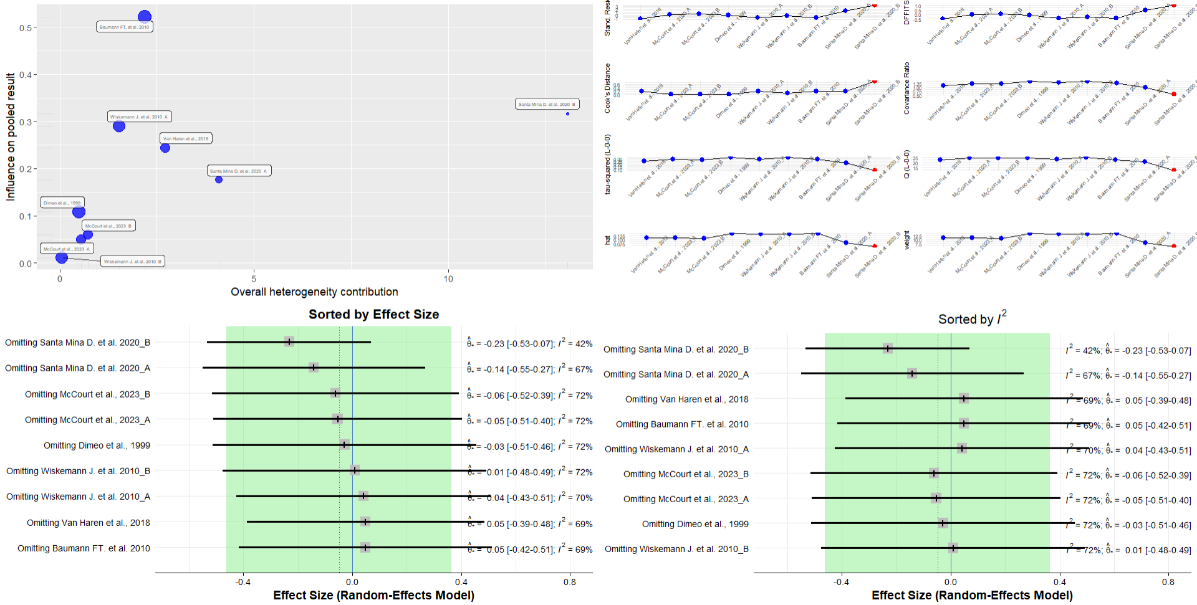
**Figure S9 – Sensitive analysis fatigue**

**Figure S10– Sensitive analysis fatigue**


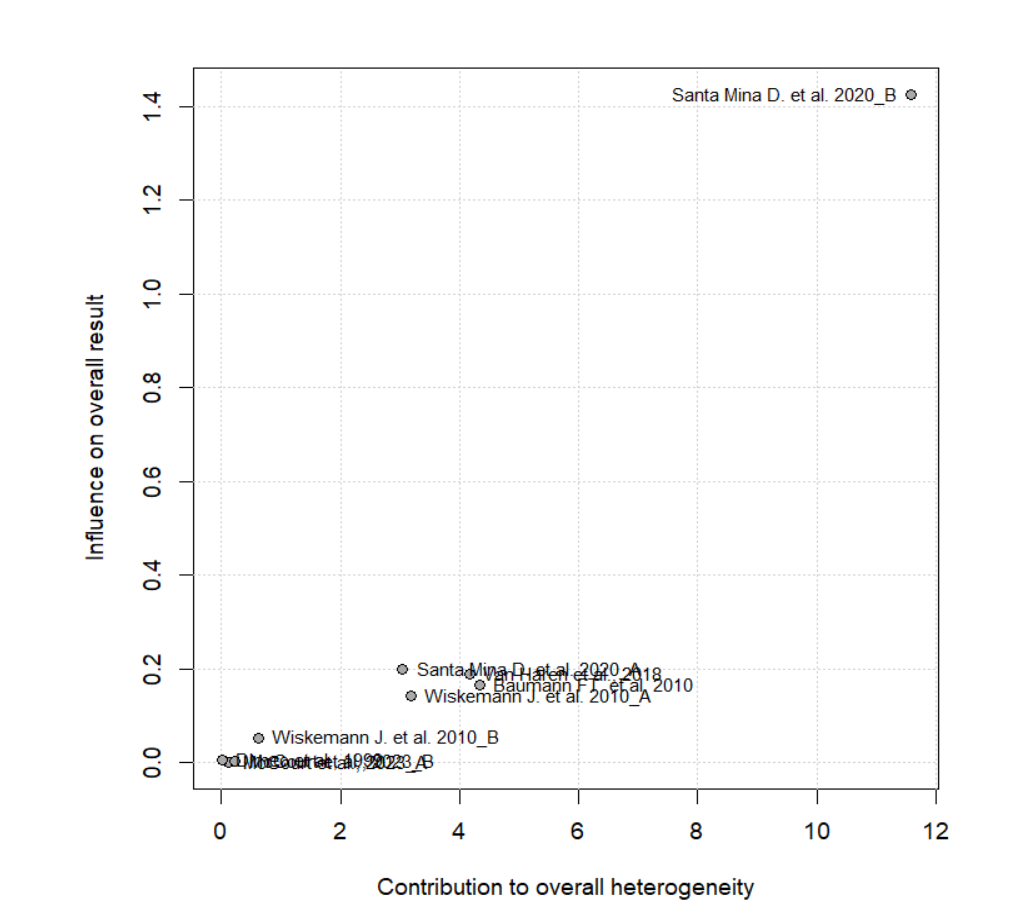


**Figure S11 – Funnel plot handgrip**


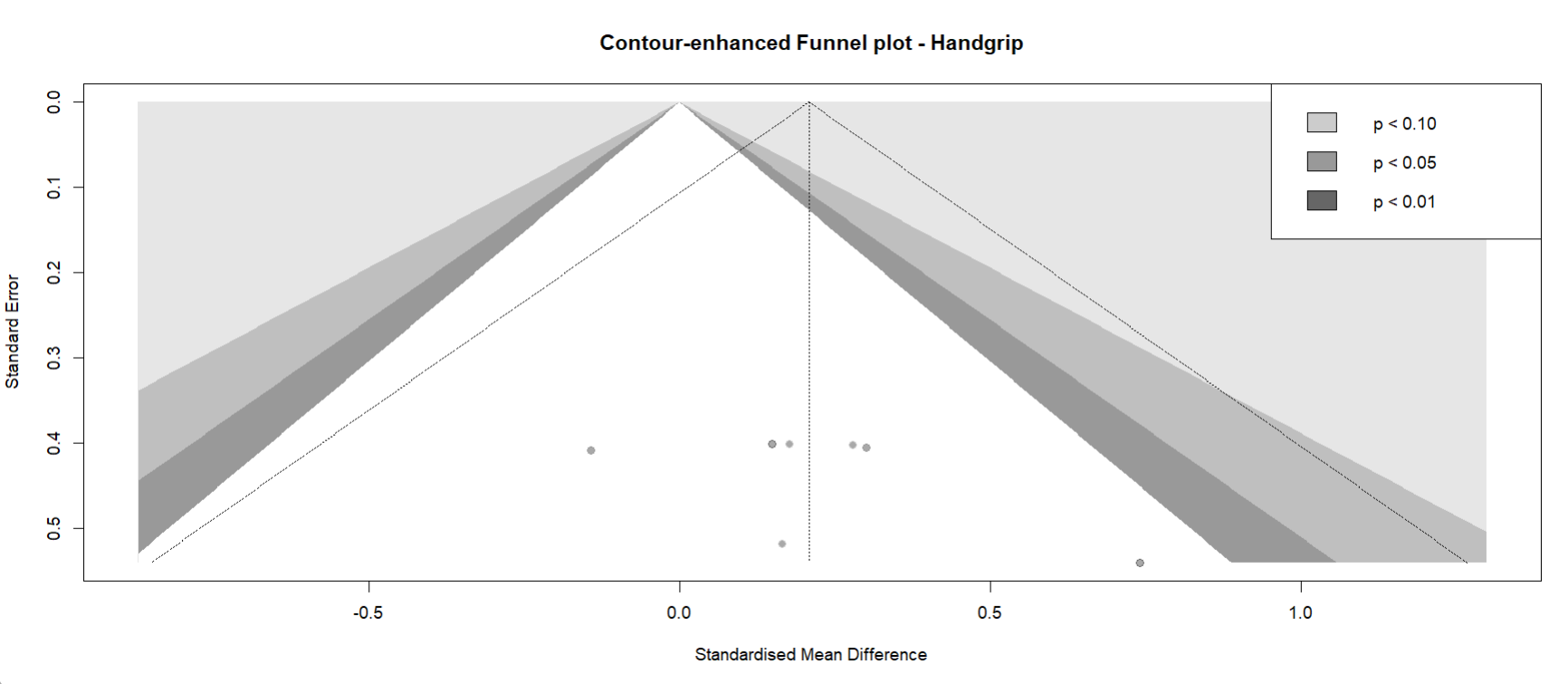


**Figure S12 - Subgroup analysis handgrip: type of transplant**


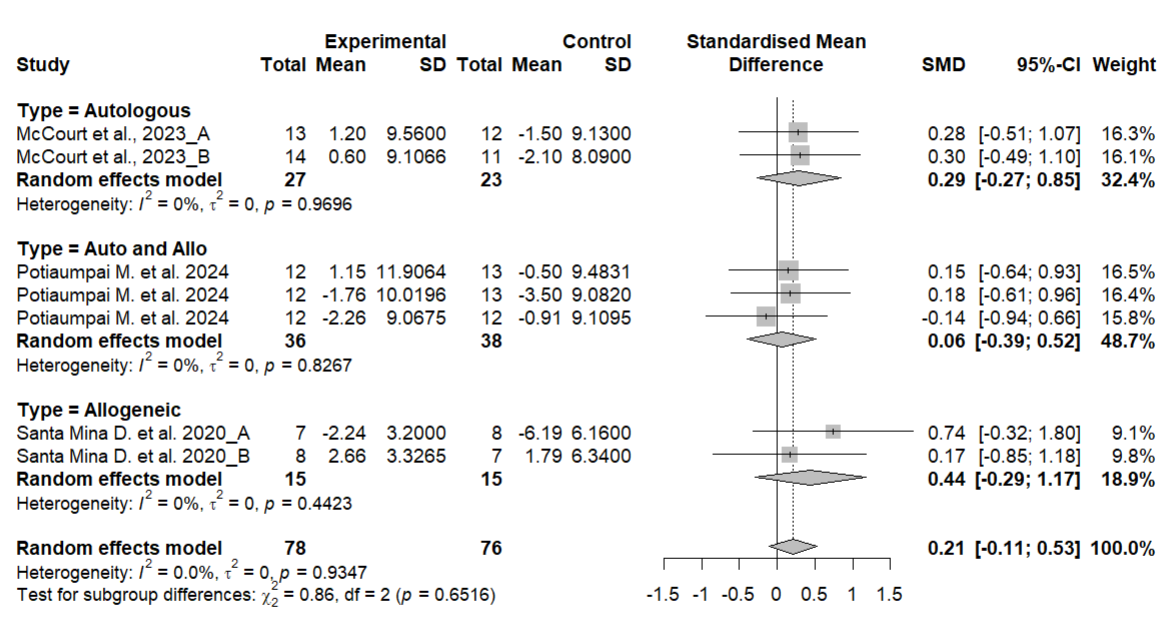


**Figure S13 - Subgroup analysis handgrip: type of exercise**


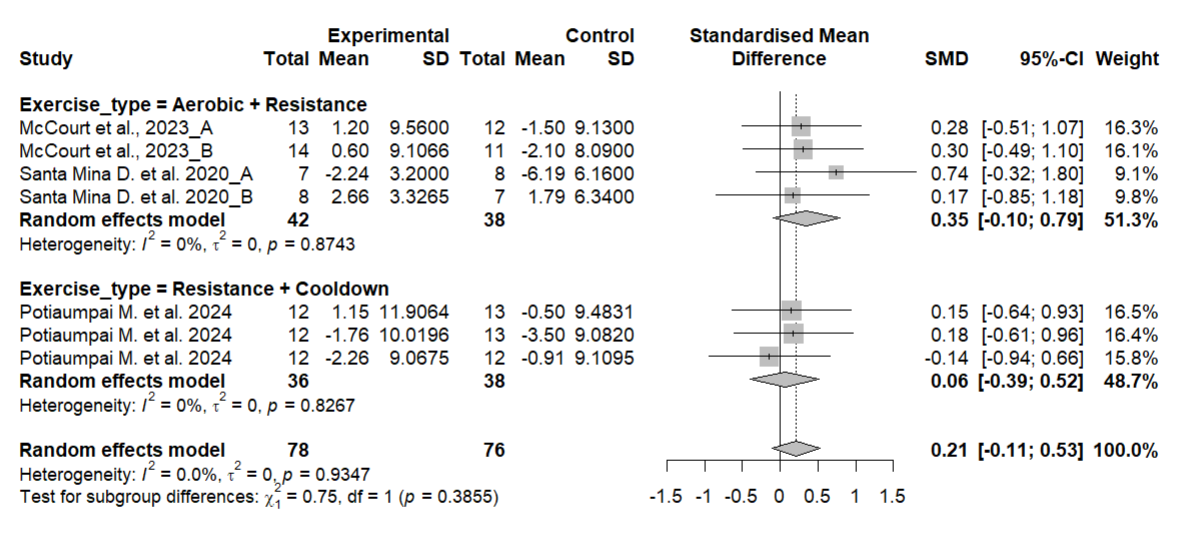


**Figure S14 – Funnel plot 30CS**


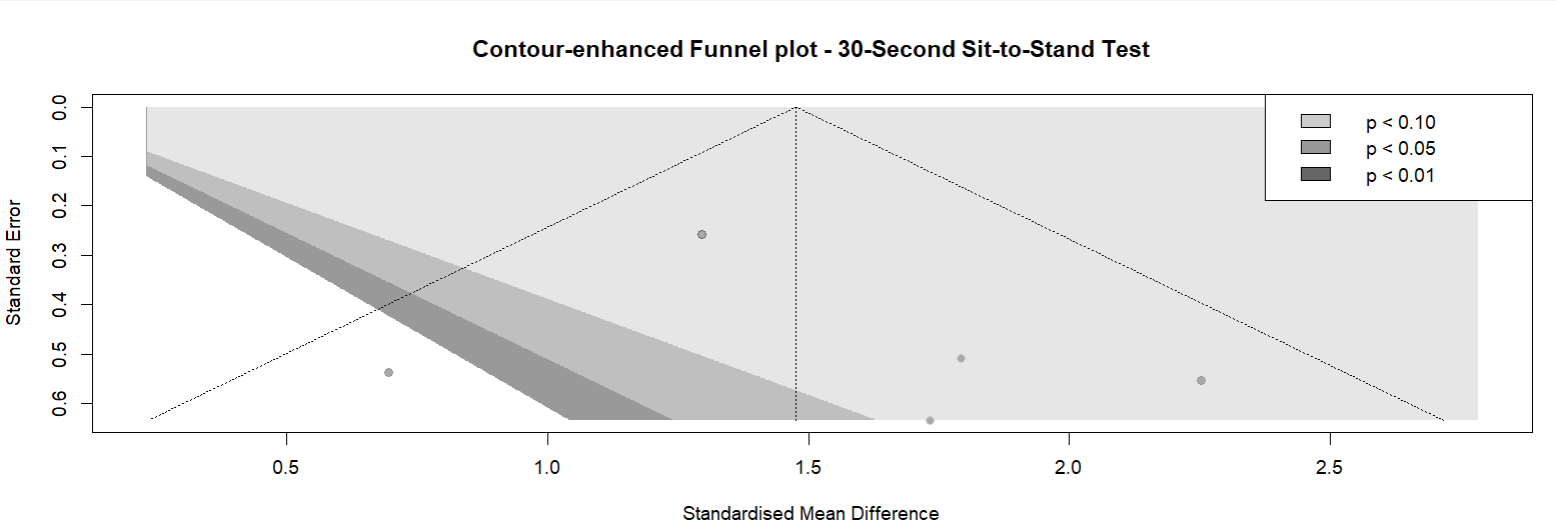


**S15. Sensitivity analysis**

Quality of life. Removing each study one at a time did not substantially alter the direction or significance of the overall findings. When McCourt et al., 2023_A (23] was excluded, the pooled effect size increased slightly and became statistically significant (SMD = 0.28; 95% CI: 0.031 to 0.53; p = 0.028), while heterogeneity remained null (I² = 0%). This suggests that this particular study may slightly attenuate the overall effect size. However, no single study significantly altered the magnitude or direction of the pooled estimate, indicating the robustness of the findings.

6MWT: The pooled SMD remained statistically significant and within a similar range regardless of which study was omitted. Effect sizes ranged from 0.36 to 0.50, with all corresponding confidence intervals excluding zero except when omitting McCourt et al., 2023_B [23]. Heterogeneity values (I²) varied from 0% to 36.9%, with tau² consistently low across iterations. These findings indicate that no single study had a disproportionate influence on the overall result, supporting the stability of the meta-analytic estimate.

Fatigue***:*** sensitivity analysis was conducted excluding the study by Santa Mina D. et al. 2020 [29]. The exclusion of this study changed the significance of the overall effect, showing that it changed from (SMD = -0.05; 95% CI: -0.46 to 0.36; n = 337; Z = 0.23; p = 0.819) to (SMD = -0.31; 95% CI: -0.57 to -0.06; n = 307; Z = 2.43; p = 0.015), indicating that this study had a considerable impact on the heterogeneity (incresasing it) and overall results of the meta-analysis. When excluded, the pooled effect became statistically significant, suggesting that the findings related to fatigue should be interpreted with caution.

Handgrip: SMD ranged from -0.23 to 0.05 when each study was excluded in turn, with no significant changes in the overall effect observed (p-values ranged from 0.13 to 0.97). The pooled effect remained small and non-significant (SMD = -0.05; 95% CI: -0.46 to 0.36; p = 0.82), with substantial heterogeneity persisting (I² between 41.9% and 72.4%). These findings indicate that no single study disproportionately influenced the meta-analysis results, confirming the stability of the effect estimate despite the high heterogeneity observed.

30CST: Sequential exclusion of each study showed that the SMD remained consistently significant with a large effect size, ranging from 1.33 to 1.61, with confidence intervals not crossing the null effect line (p < 0.0001 in all cases). Heterogeneity (I²) varied between 0% and 38.3%, and between-study variance (tau²) was low to moderate, indicating that no single study had a substantial influence on the overall results. These findings confirm the robustness and consistency of the positive effect of post-transplant exercise on functional capacity assessed by the 30CST.

**Figure S16 – Meta-regression of weeks of intervention on fatigue**


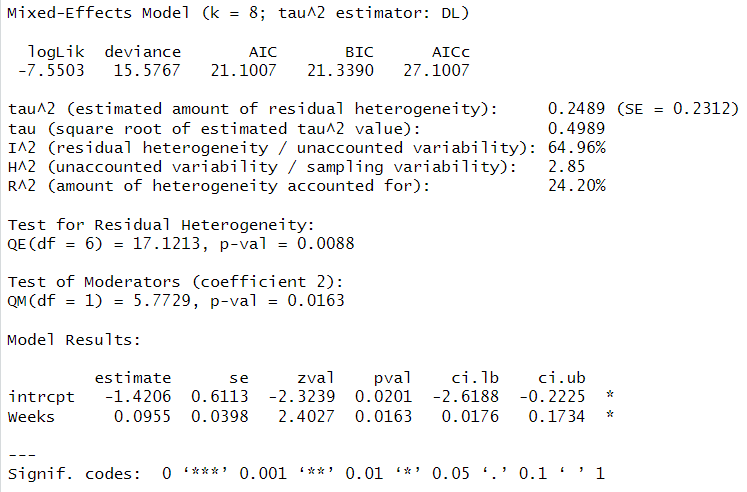


**Figure S17 - Bubble plot of meta-regression: Weeks of intervention vs. fatigue (bubble size = study weight)**


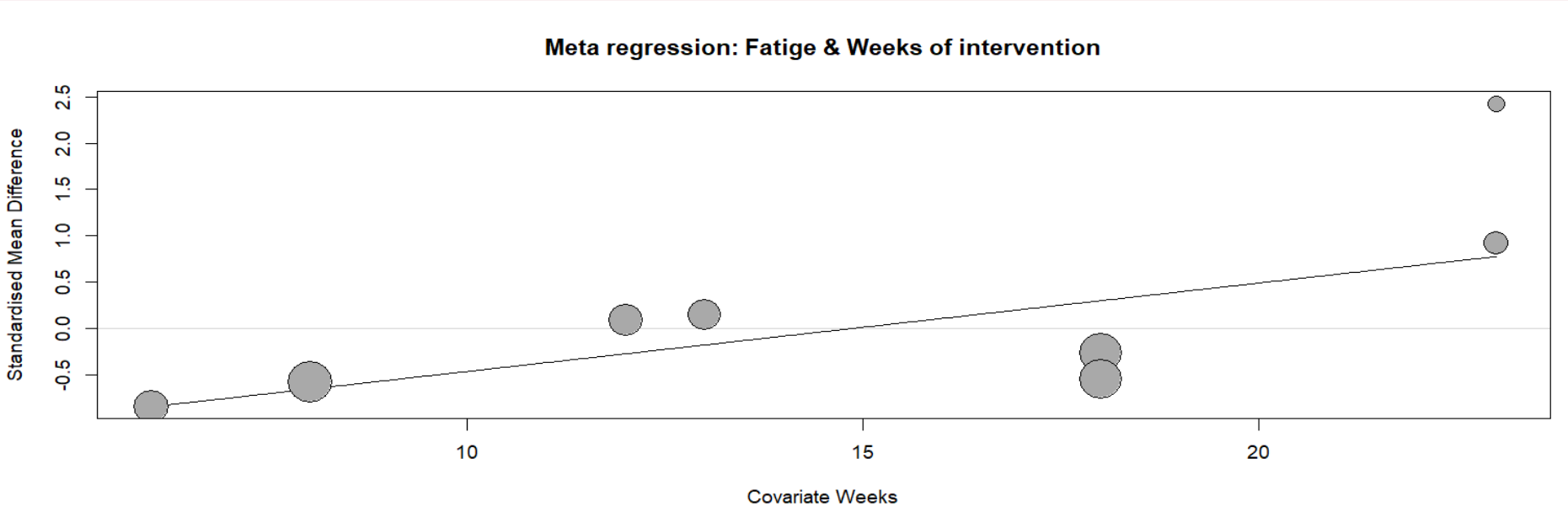

Supplement: Supplementary file 2 — (DOCX 2.45 MB) [file 520_2025_10194_MOESM2_ESM.docx]
